# Spontaneous regression of a congenital high-grade glioma—a case report

**DOI:** 10.1093/noajnl/vdab120

**Published:** 2021-08-25

**Authors:** Maria Riedmeier, Annika Stock, Jürgen Krauß, Felix Sahm, David T W Jones, Dominik Sturm, Christof M Kramm, Matthias Eyrich, Christoph Härtel, Simon Schlegel, Paul Gerhardt Schlegel, Camelia-Maria Monoranu, Verena Wiegering

**Affiliations:** 1 Department of Pediatric Hematology, Oncology and Stem Cell Transplantation, University Children’s Hospital, University of Würzburg, Würzburg, Germany; 2 Department of Neuroradiology, University Hospital, University of Würzburg, Würzburg, Germany; 3 Department of Pediatric Neurosurgery, University Children’s Hospital, University of Würzburg, Würzburg, Germany; 4 Department of Neuropathology, University Hospital, University of Würzburg, Würzburg, Germany; 5 Comprehensive Cancer Centre Mainfranken, University of Würzburg Medical Centre, Würzburg, Germany; 6 Department of Neuropathology, University Hospital Heidelberg, Heidelberg, Germany; 7 Clinical Cooperation Unit Neuropathology, German Cancer Consortium (DKTK), German Cancer Research Center (DKFZ), Heidelberg, Germany; 8 Hopp Children’s Cancer Center (KiTZ), Heidelberg, Germany; 9 German Cancer Research Center (DKFZ), Heidelberg, Germany; 11 Division of Pediatric Neurooncology, German Cancer Consortium (DKTK), German Cancer Research Center (DKFZ), Heidelberg, Germany; 12 Division of Pediatric Hematology and Oncology, University Medical Center Göttingen, University of Göttingen, Göttingen, Germany; 13 Bavarian Cancer Research Center (BZKF), University of Würzburg, Würzburg, Germany; 14 Semmelweis University, Budapest, Hungrary

**Congenital anaplastic astrocytomas and glioblastomas (cGBMs) are rare tumors of the central nervous system.** **^1^ Most cGBMs are histologically indistinguishable from GBM in older children and adults but may have a more favorable outcome, suggesting biological and molecular differences. We describe a case of spontaneous regression of a cGBM with molecular evidence of a CLIP2–MET fusion.**

## Case Presentation

A male preterm neonate of 36 gestational weeks was born by Caesarean section to a 34-year-old mother. Routine prenatal ultrasound at 35 weeks of gestation had demonstrated a hydrocephalus internus as a consequence of an intracranial hemorrhage.

The newborn presented with good postnatal adaptation (APGAR score 8/10/10) and was breathing spontaneously, with stable vital parameters. Abnormal clinical findings included a macrocephalus (head circumference: 41 cm, >99th percentile, *z*-score 5.7) and a congested and large fontanelle with gaping cranial sutures. Laboratory diagnostics showed a heterozygous Factor V Leiden mutation, but otherwise no evidence of thrombophilia, coagulation disorder, or blood count abnormalities. Newborn screening tests were normal.

## Clinical Course

Due to rapidly increasing hydrocephalus internus detected in a tightly scheduled ultrasound examination, a Rickham reservoir was inserted on day 3 postpartum with the cerebrospinal fluid being collected daily. Initially, the patient was irritable to touch and with recurring vomiting but no episodes of seizures. Since diagnostics of infections, pathogens, and inflammatory markers remained negative, posthemorrhagic meningitis was postulated. Analgetic treatment led to an improvement in symptoms.

**Figure 1.**
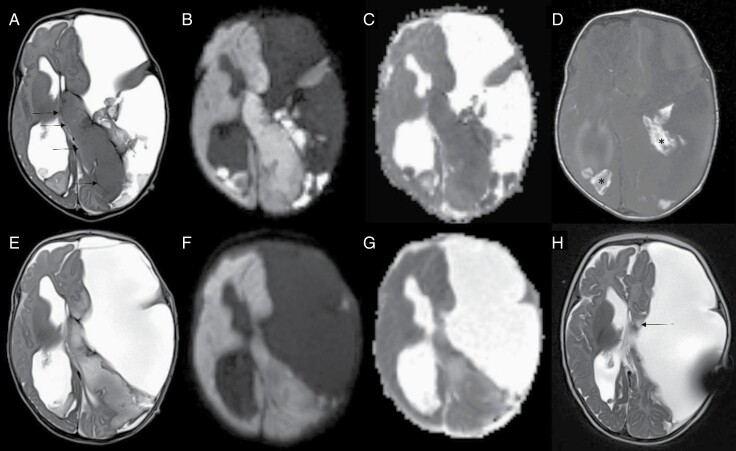
(A–D) First MRI. (A) Axial T2-weighted image shows homogeneous tumor tissue, mainly involving the left frontal and temporal lobe (black arrows), which is of isointense signal intensity compared with the cerebral cortex. Massive posthemorrhagic enlargement of the subdural space is visible on the left. (B and C) Diffusion-weighted images show a slight diffusion restriction as a sign of high cellularity. (D) T1-weighted image shows clots of methemoglobin (*). (E–G) Follow-up MRI at day 24. (E) T2-weighted image shows a substantial increase in the signal intensity of the tumor. (F and G) Diffusion-weighted images show decreasing diffusion restriction. (G) Tumor tissue is now partially hyperintense on apparent diffusion coefficient (ADC) compared with brain parenchyma. Imaging changes in E–G were interpreted as a change from high to lower cellularity. (H) At 5 months after diagnosis, only small, stable tumor residues could be found (black arrow).

At the age of 4 weeks, MRI showed a homogeneous tumor, with morphological signs of high-grade glioma, mainly involving the left frontal and temporal lobe, and a massive posthemorrhagic enlargement of the subdural space on the left (Figure 1A–D). Three days later, the patient underwent endoscopic resection of the hematoma and tumor biopsy.

**Figure 2.**
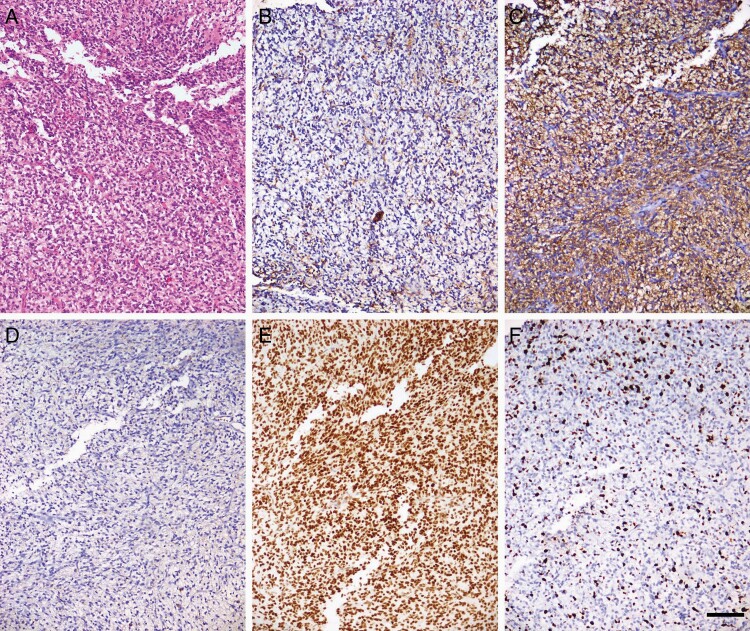
High-grade glioma with high cellularity, brisk mitotic activity, and microvascular proliferation without necrosis, H&E staining (A). Focal expression of glial fibrillary acidic protein (GFAP) (B), strong expression of MAP2 (C), negativity for IDH1 (R132H) (D), retained nuclear expression of ATRX (E), and proliferation index Ki-67 with an average of 20% (F) (scale bar 100 µm; magnification 100×).

**Figure 3.**
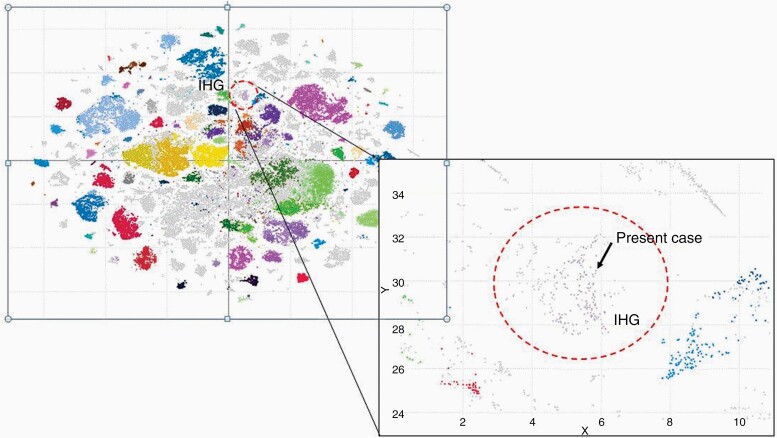
Methylation profile of the tumor (Sentrix ID: 204273190021_R02C01) in relation to the big reference dataset of tumors. tSNE visualization using the Fit-SNE algorithm, using the top 50 000 most differentially methylated CpG sites from the methylation arrays. More than 90 000 DNA methylation samples are represented, including over 60 000 brain tumors. The coloring of the reference groups is according to Capper et al. *Nature* 2018. This further shows that the tumor has the greatest similarity with the “Infantile Hemispheric Glioma” tumor group (IHG). The IHG group (colored in light purple) is circled with a red dashed line and labeled. The present case is marked with a black arrow, as shown in more detail on the zoomed-in region.

Histopathological evaluation revealed a high-grade glioma with high cellularity, brisk mitotic activity, and microvascular proliferation without necrosis. The immunophenotype supported the diagnosis, with a focal expression of glial fibrillary acidic protein, strong expression of MAP2, and negativity of neuronal markers such as synaptophysin and neurofilaments. Nuclear expression of ATRX was retained and immunoreactivity for mutant IDH1 (R132H) was negative. The proliferation rate of tumor cells, estimated using the Ki-67 index, was high (~20%; Figure 2). DNA methylation array classified the tumor as an infantile hemispherical glioma, with evidence of a CLIP2–MET fusion detected by next-generation sequencing (Figure 3). The CLIP2–MET fusion protein is known to activate the MAPK pathway, one of the driver pathways responsible for the oncogenesis of glioneuronal tumors.^2^

In accordance with the parents’ preferences, any therapeutic decision was postponed and a watch-and-wait strategy with tightly scheduled MR imaging was chosen. Justification for this strategy was the massive brain defect with unclear neurological outcome, limited overall prognosis in cGBM, and the uncertainty of appropriately applying chemotherapy in this very young age group.

Surprisingly, follow-up MRI at 10 weeks showed a significant reduction of the tumor volume, and at the age of 6 months, only a small residual tumor was detected (Figure 1E–H).

Clinical examination at the age of 10 months showed a fine-beat pendular nystagmus with the convergence of the left eye and a discreet movement reduction on the right side. The boy presented with intermittent suspicious movements compatible with focal seizures corresponding to epileptic activity by electroencephalogram.

## Discussion

Recent molecular studies have revealed biological subgroups of infant gliomas with different genetic alterations and outcome.^3,4^ The present case belongs to a group of hemispheric RTK-driven gliomas harboring ALK/ROS1/NTRK/MET alterations. Two reported cases of high-grade gliomas from this group displayed lower-grade histology upon the second resection, suggesting that such tumors may have the potential to differentiate and slow their growth over time. A potential mechanism involved in spontaneous differentiation of gliomas harboring ALK/ROS1/NTRK/MET alterations is oncogene-induced senescence. This has been described for infantile hemispheric gliomas type I, which may fit into our DNA methylation analysis (Figure 3).^3^ Furthermore, as seen in common infant tumors that harbor ALK, inherent maturation as part of normal development is an alternative explanation of the morphological and clinical maturation of infantile high-grade gliomas into infantile low-grade gliomas.^3^ The phenomenon of spontaneous regression of solid tumors arising during embryonal development is known for pediatric tumors, known for pediatric tumors, most prevalent in neuroblastoma.^5^

The present case, with clinical and neuroradiological signs of tumor differentiation, once more underlines the different clinical behavior of a subset of cGBMs compared with GBM in older patients and highlights the growing relevance of molecular classification of brain tumors in improving the prognostic evaluation and identification of potential therapeutic targets.^6^ Since infantile gliomas are mostly single-driver tumors, they are particularly suitable for precision-medicine treatment approaches, and especially ALK inhibitors and tyrosine kinase inhibitors may be an additional therapeutic option in the future during rapid growth phases of the tumor.

In conclusion, the clinical course of the present case is encouraging the suggestion that for some subgroups of infant gliomas careful observation—a strategy that is well accepted in neuroblastomas—may be an alternative to intense oncology treatment.
